# Population cycles emerging through multiple interaction types

**DOI:** 10.1098/rsos.170536

**Published:** 2017-09-27

**Authors:** Naoya Mitani, Akihiko Mougi

**Affiliations:** Department of Biological Science, Faculty of Life and Environmental Science, Shimane University, 1060 Nishikawatsu-cho, Matsue 690-8504, Japan

**Keywords:** cycling, predator–prey, mutualism, competition, stability, mathematical model

## Abstract

Cyclic dynamics of populations are outstanding and widespread phenomena across many taxa. Previous theoretical studies have mainly focused on the consumer–resource interaction as the driving force for such cycling. However, natural ecosystems comprise diverse types of species interactions, but their roles in population dynamics remains unclear. Here, using a four-species hybrid module with antagonistic, mutualistic and competitive interactions, we analytically showed that the system with major interaction types can drive population cycles. Stronger interactions easily cause cycling, and even when sub-modules with possible combinations of two interactions are stabilized by weak interactions, the system with all interaction types can cause unstable population oscillations. Diversity of interaction types allows to add mutualists to the list of drivers of oscillations in a focal species' population size, when they act in conjunction to other drivers.

## 1. Introduction

Regular cyclic oscillations in populations observed across many taxa have intrigued ecologists for nearly a century since Elton's studies [1]. Previous theories have proposed two fundamental mechanisms, internal and external forces, for these oscillations. The internal force includes demographic details, such as physiological changes [2,3], age-structure [4–6] and evolutionary adaptation [7–11], while the external one includes abiotic environmental fluctuations, such as stochasticity [12–14] seasonality [15–17] and species interactions [18–22].

Historically, a major species interaction which highly attracted attention was antagonism, including predator–prey interaction, due to its inherent tendency to oscillate [23]. Real ecosystems, however, comprise diverse species, suggesting that multiple interactions can interactively cause cycling [24,25]. Although these studies focused on antagonistic interactions and contributed to our understanding of how interactions among multiple species are related to population cycles, other dimensions of the ecosystem remain to be studied.

Ecological communities comprise not only diverse species but also diverse interspecific interactions that link them, such as predation, parasitism, competition and mutualism. Recent theoretical studies have shown that such diversity in species and interaction types plays a major role in ecological communities through synergism [26–30], giving rise to a positive relationship between complexity and stability [26,28–30]. By contrast, there is little understanding regarding how multiple interactions affect the population dynamics in low-dimensional systems.

A recent study examined the effects of a mixture of different interaction types on population dynamics in a community module (i.e. a simple system with few species) [31]. It presented a mathematical model of a three-species hybrid module, the dynamics of which are driven by antagonistic and mutualistic interactions. The model showed that the hybridity of the two interaction types tended to destabilize the system and lead to population oscillations with large amplitudes, despite the subsystems with either interaction type being globally stable [31]. However, only two interaction types were considered in that model, leaving an unanswered question of what effect, if any, other interaction types and mixing have on population dynamics.

Here, we introduce another major interaction type, competitive interaction, into the hybrid module with antagonistic and mutualistic interactions and show that population cycles can also emerge through multiple interaction types. In natural communities, a certain species usually has different interaction types with different interaction partners. For example, in forest communities, plants interact with herbivores, pollinators, parasitic fungi, mutualistic fungi and competing plants, forming antagonistic, mutualistic and competitive interactions [32,33]. Similarly, in freshwater communities, protists with mutualistic partners such as chlorella interact with predators and competing protists [34–36]. Despite the universality of hybrid modules with the major interaction types in natural communities, their role in population dynamics remains less understood.

In this study, using a four-species model that incorporated antagonistic, mutualistic and competitive interactions, we analysed the effects of interaction-type diversity on population dynamics. We analytically show that population cycles emerging through multiple interaction types is a general feature of the simple module. Moreover, even when sub-modules with combinations of two interaction types are stabilized by weak interactions, we show that the system with all interaction types can cause population oscillations of large amplitudes, suggesting that mutualists can be a driver of population cycles through the interactions with other major interaction types.

## 2. Model

**Figure 1.**
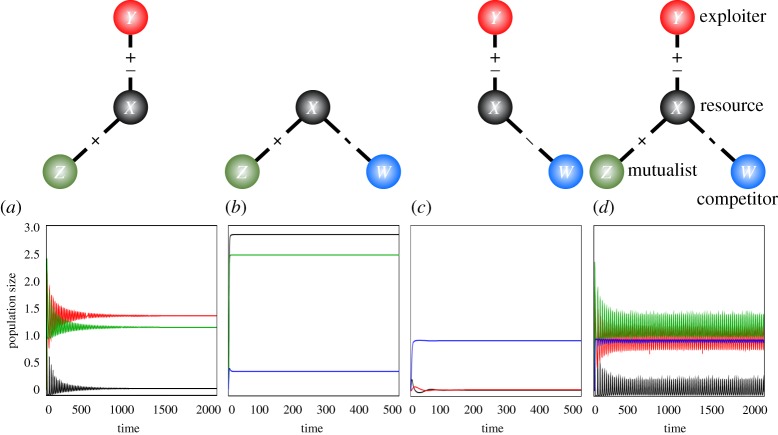
Dynamics of population sizes. Illustrations along the upper side of the panels indicate the community modules that cause each population dynamics. The signs within the linking lines indicate the antagonistic (±), mutualistic (+) and competitive (–) interaction types. Colours indicate different species. Parameter values are: *r_X_* = *r_Z_* =* r_W_* = 1, *g =* 0.25, *d = *0.05, *h_X _= h_Z _=* 1, *a = *1.8, *u =* 3, *v =* 2, *α *= 0.2 and *β = *0.7. We chose this to satisfy the condition under which the sub-modules are under stable equilibrium, but the mixture of such modules causes instability (in equations (3.1)–(3.4)). Even if the strong symmetrical parameter values are relaxed, the same hybrid effect can be found if these inequalities are balanced.

Consider a simple four-species community where a resource species interacts with three other species: an exploiter, a mutualist and a competitor. This four-species system is the simplest system to comprise the major types of interaction: antagonistic, mutualistic and competitive. It may also reflect a prevalent ecological module consisting of a plant, its herbivore, a pollinator or seed disperser and a competing plant. We can extend the simplest model community with antagonistic and mutualistic interactions [31] to one with competitive interaction (figure 1). The population dynamics can then be described by the following differential equations:

2.1 $$\begin{array}{rl} \frac{\text{d}X}{\text{d}t} & =\left(r_{X}-X-\beta W-aY+\frac{uZ}{h_{Z}+Z}\right)\;X, \end{array}$$

2.2 $$\begin{array}{rl} \frac{\text{d}W}{\text{d}t} & =(r_{W}-W-\alpha X)W, \end{array}$$

2.3 $$\begin{array}{rl} \frac{\text{d}Y}{\text{d}t} & =(gaX-d)Y \end{array}$$

2.4 $$\begin{array}{rl} \;\text{and}\;\frac{\text{d}Z}{\text{d}t} & =\left(r_{Z}-Z+\frac{vX}{h_{X}+X}\right)\;Z, \end{array}$$

where *X*, *W*, *Y* and *Z* are the population levels of the resource species, competitor, exploiter and mutualist, respectively. The other parameters are as follows: *r_i_* is the *per capita* growth rate of the resource (*i = X*), competitor (*i = W*) and mutualist (*i = Z*); *α* and *β* are the competition coefficients (which are implicitly normalized by self-regulations assumed to be without loss of generality); *a* is the capture rate, defined as the *per capita* rate at which an exploiter captures the resource; *g* is the conversion efficiency, which relates the birth rate of the exploiter to its resource consumption; *d* is the death rate of the exploiter; *u* and *v* are the maximum benefits of the mutualistic interaction; and finally, *h_X_* and *h_Z_* are the half-saturation constants of the hyperbolic functional response. The strength of self-regulation for the mutualist is assumed to be 1 for analytical simplicity without loss of generality.

We focus on a case where mutualism is facultative (*r_i_* > 0). This assumption allows globally stable coexistence of both predator–prey and mutualistic pairs in isolation [31] and in the absence of competitor. However, the coupling of such globally stable systems can cause a stable limit cycle, suggesting a destabilizing effect of interaction-type diversity on population dynamics [31]. Here, we examine whether this prediction is robust following the introduction of competition as another major interaction type.

## 3. Results

**Figure 2.**
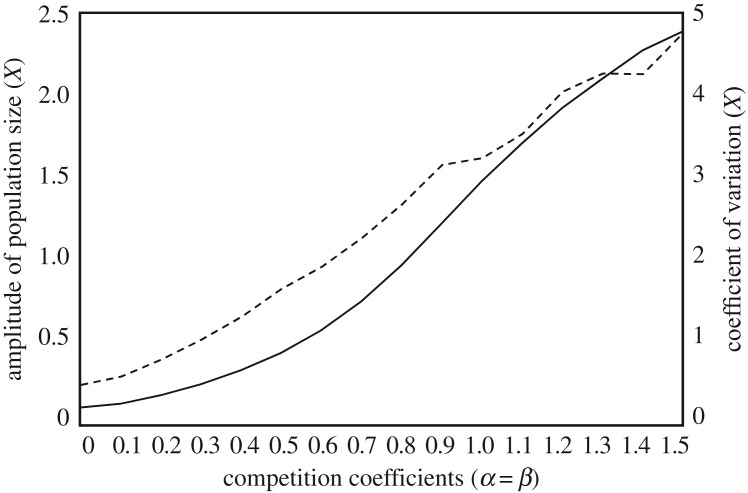
Effects of interspecific competition on the amplitudes and coefficient of variation of population oscillations. The amplitude (solid line) and coefficient of variation (dashed line) of population oscillation were calculated after using the dynamic approach to asymptotic behaviours, with varying competition coefficients. The resource species is used as the index of the amplitude. The tendency was the same regardless of the species used. Parameter values are the same as in figure 1 except for *a =* 2.5.

The analysis showed a major effect of competitive interaction on the population dynamics of the hybrid module with antagonistic and mutualistic interactions (the AM module). The invasion of a competitor tended to destabilize the hybrid community, amplifying population oscillations caused by the hybridity of antagonistic and mutualistic interactions. With increasing strength of competitive interaction, the amplitudes of population oscillations also increased (figure 2). Even when the AM module was stable in isolation, adding competitive interaction caused a limit cycle to occur (figure 1). Interestingly, in an extreme case where all sub-modules—AM, AC (antagonism-competition) and MC (mutualism-competition)—were stable in isolation, such a limit cycle could still occur (figure 1). In these instances, each system was globally stable when present with one interaction type, suggesting that population oscillations can be induced by hybridity of stable sub-modules with different interaction types.

How does the hybridity of stable sub-modules cause population oscillations? Local stability analysis can help with the understanding of this phenomenon. First, we focused on the stability of AM and AC modules because these modules inherently cause limit cycles (using numerical simulations, we confirmed that only the MC module did not cause population oscillations). Then, we examined the stability of the full model (equations (2.1)--(2.4)) and analysed the local stability of each system by linearizing the dynamics near the non-trivial equilibrium. We judged the local stability by whether the characteristic equation of their Jacobian matrix satisfied the Routh–Hurwitz criteria.

Without a competitor (*W* = 0), limit cycle never occurred in the AM module, and all species coexisted stably when the following criterion was met [31]:

3.1 $$Y^{*}>\frac{Z^{*}(X^{*}+Z^{*})(M-1)}{ad},$$

where X* = *d*/*ga*, Y* = (1/*a*){*r_X_* – X* + uZ*/(*h_Z_* + Z*)}, Z* = *r_Z_* + vX*/(*h_X_* + X*) and *M = uvh_X_h_Z_*/ (*h_X _*+ X*)^2^(*h_Z _*+ Z*)^2^. The asterisk indicates the non-trivial equilibrium. Here, *M* is the ‘antagonistic and mutualistic effect’ because it becomes large by efficient exploitation (large *a*) and sufficient mutualism (large *u* and/or *v*). Equation (3.1) clearly shows that weaker antagonistic and mutualistic effects (*M < *1) do not cause population cycles in the AM module.

In a similar way, without mutualism (*Z *= 0), limit cycle never occurred in the AC module, and all species coexisted stably when the following criterion was met (see electronic supplementary material, S1):

3.2 $$Y^{*}>\frac{W^{*}(X^{*}+W^{*})(\alpha \beta -1)}{ad},$$

where W* =* r_W _*− αX* and Y* = (1/*a*)(*r_X _*– X* − βW*). This implied that weaker interspecific competition (*αβ *< 1) did not cause population cycles in the AC module. These results show that sub-modules with antagonistic interactions (the AM and AC modules) did not cause stable limit cycles when antagonistic, mutualistic or competitive interactions were weak. Thus, population cycles may be caused by a coupling effect of sub-modules. Indeed, we show that stability with the three interaction types can be broken in the hybrid module even when the equilibria in each antagonistic sub-module are locally stable (the AM and AC modules).

Local stability analysis indicated that either of the following conditions was necessary for cycles to occur in the full model (electronic supplementary material, S1):

3.3 $$Y^{*}<L(\alpha \beta +M-1),$$

or

**Figure 3.**
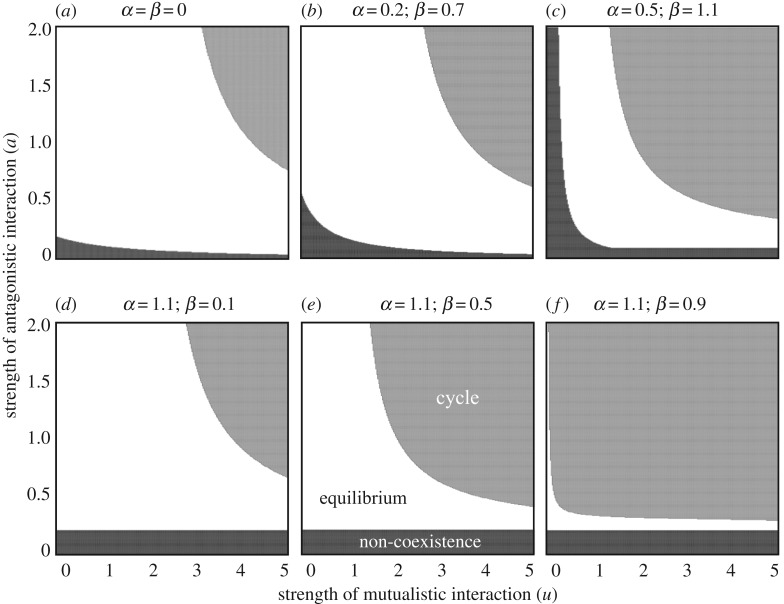
Parameter regions indicating stability of the coexistence equilibrium. Coexistence is impossible in the black region. However, the coexistence equilibrium is stable in the white region and unstable (a limit cycle occurs) in the grey region. The values of competition coefficients (non-free parameters) are shown above the panels. Parameter values are the same as in figure 1.

3.4 $$Y^{*}<L(W^{*}+Z^{*})\;\left\{\frac{\alpha \beta -1}{Z^{*}}+\frac{M-1}{W^{*}}-\frac{1}{X^{*}}\right\},$$

where Y* = (1/*a*){*r_X_* − X* − βW* + uZ*/(*h_Z_* + Z*)} and L = X*W*Z*/{*da*(W* + Z*)}. Equation (3.3) suggests that the equilibrium can be locally unstable if the sum of the antagonistic and mutualistic effect (*M*) and competition effect (*αβ*) are greater than 1, even when each interaction effect is weak or less than 1 (*M* < 1 and *αβ* < 1). This prediction is also supported by numerical analysis (figure 3). In addition, equations (3.3) and (3.4) show that larger effects of interspecific interaction effects (*M*, *αβ* > 1) tend to destabilize the system (electronic supplementary material, S2). In an extreme case where the exploitation rate (*a*) was very large, we proved that the equilibrium was always unstable (electronic supplementary material, S1).

## 4. Discussion

Interaction-type diversity has attracted attention in the field of community ecology [26,37–42]. However, little is understood about its role in population dynamics. In this study, we analytically showed that interaction-type diversity was associated with inherent population oscillations in a simple community module. To allow stability in the system, sufficiently weak interaction strengths are necessary for all interaction types. However, even if each interaction is weak enough to stabilize sub-modules without another interaction type, the stability may be broken by bonding of the three interaction types, resulting in population oscillations with large amplitudes. This provides a novel role of mutualist in driving population cycles through the interactions with other multiple interaction types.

Population cycles caused by multiple interaction types result from the indirect effects of the exploiter on mutualist or competitor through a resource species. The mutualist indirectly increases the exploiter through an increase in the mutualistic partner resource species. Then, the increase in the exploiter indirectly increases the competitor through higher exploitation of the resource species and simultaneously, but also indirectly, decreases the mutualist. Through these indirect effects, resource species are reduced to a large degree, which in turn reduces the level of the exploiter and causes the cycle to begin again.

The present theory predicts that three major interaction types under study can cause unstable population cycles with large amplitudes and does not allow species to stably coexist when interactions are strong. This prediction is supported by an earlier model with a large ecological network. In their research, Mougi and Kondoh demonstrated that interaction-type diversity can stabilize larger communities with many species, giving rise to a positive complexity–stability relationship provided that each species allocates their interaction efforts separately to each interacting partner species [26,28,29]. This suggests that unstable population oscillations are likely to occur in smaller systems, because the interaction strengths become stronger in such smaller systems, given the interaction effort allocation. This biological viewpoint reinforces the prediction of population cycles due to multiple interaction types in small communities.

There are three possibilities as to why cycles are caused by species interaction. First, as is classically known, strong interspecific competition can cause cycles by combining with antagonistic interactions [43]. Second, a strong coupling of antagonistic and mutualistic interactions could be involved [31]. Third, cycles may only emerge by mixing all three interaction types. Although predator–prey cycles have been a key mechanism in population cycles, we propose a new mechanism for population cycles, namely mutualist induced cycles under the existence of multiple interaction types.

Our prediction from the simple model in this study is supported by a more mechanistic but analytically intractable model [44]. In a similar system construct to ours, several specific functional forms were assumed: type III functional response of the exploiter to the resource; mutualistic interaction with density-dependent costs and benefits; obligate mutualism; density-dependent mortality of the exploiter and a protection effect by mutualistic fungi for the resource plant against the enemy. The hybrid system also shows population cycles with large amplitudes, but it is unclear whether the cycles are caused by multiple interaction types, because the modules with antagonistic or mutualistic interactions are inherently unstable.

The present theory sheds light on the mechanisms underlying population cycles. One such mechanism includes higher dimensional systems with multiple species beyond a classical pairwise consumer–resource interaction [45]. Another dimension, interaction-type diversity, may aid in further understanding the population cycles.

## Supplementary Material

Local stability analysis of the coexistence equilibrium

## Supplementary Material

Supplemental figure

## Supplementary Material

Mathematica code that can produce the all figures

## Data accessibility

Mathematica code supporting the results in this study is available as electronic supplementary material, S3.

## References

[1] Elton CS 1924 Periodic fluctuations in the numbers of animals: their causes and effects. J. Exp. Biol. 2, 119–163.

[2] Ginzburg L, Colyvan M. 2003 Ecological orbits: how planets move and populations grow. New York, NY: Oxford University Press.

[3] de Roos AM, Galic N, Heesterbeek H 2009 How resource competition shapes individual life history for nonplastic growth: ungulates in seasonal food environments. Ecology 90, 945–960. (doi:10.1890/07-1153.1) 19449690 10.1890/07-1153.1

[4] Claessen D, de Roos AM, Persson L 2000 Dwarfs and giants: cannibalism and competition in size-structured populations. Am. Nat. 155, 219–237. (doi:10.1086/303315) 10686162 10.1086/303315

[5] Murdoch W, Kendall B, Nisbet R, Briggs C, McCauley E, Bolser R 2002 Single-species models for many-species food webs. Nature 417, 541–543. (doi:10.1038/417541a) 12037520 10.1038/417541a

[6] de Roos AM, Persson L. 2013 Population and community ecology of ontogenetic development. Princeton, NJ: Princeton University Press.

[7] Abrams PA 2000 The evolution of predator-prey interactions: theory and evidence. Annu. Rev. Ecol. Syst. 31, 79–105. (doi:10.1146/annurev.ecolsys.31.1.79)

[8] Yoshida T, Jones LE, Ellner SP, Fussmann GF, Hairston NG Jr 2003 Rapid evolution drives ecological dynamics in a predator–prey system. Nature 424, 303–306. (doi:10.1038/nature01767) 12867979 10.1038/nature01767

[9] Mougi A, Iwasa Y 2010 Evolution towards oscillation or stability in a predator–prey system. Proc. R. Soc. B 277, 3163–3171. (doi:10.1098/rspb.2010.0691) 10.1098/rspb.2010.0691 PMC2982064 20504808

[10] Mougi A, Iwasa Y 2011 Unique coevolutionary dynamics in a predator–prey system. J. Theor. Biol. 277, 83–89. (doi:10.1016/j.jtbi.2011.02.015) 21354181 10.1016/j.jtbi.2011.02.015

[11] Mougi A 2012 Predator-prey coevolution driven by size selective predation can cause anti-synchronized and cryptic population dynamics. Theor. Popul. Biol. 81, 113–118. (doi:10.1016/j.tpb.2011.12.005) 22212374 10.1016/j.tpb.2011.12.005

[12] Nisbet R, Gurney W. 1982 Modelling fluctuating populations. Princeton, NJ: Blackburn Press.

[13] Royama T. 1992 *Analytical population dynamics*. Population and community biology series. London, UK: Chapman & Hall.

[14] Kendall BE 2001 Cycles, chaos, and noise in predator–prey dynamics. Chaos Sol. Fract. 12, 321–332. (doi:10.1016/S0960-0779(00)00180-6)

[15] King A, Schaffer W 2001 The geometry of a population cycle: a mechanistic model of snowshoe hare demography. Ecology 82, 814–830. (doi:10.1890/0012-9658(2001)082[0814:TGOAPC]2.0.CO;2)

[16] Nelson W, Bjørnstad O, Yamanaka T 2013 Recurrent insect outbreaks caused by temperature-driven changes in system stability. Science 341, 796–799. (doi:10.1126/science.1238477) 23907532 10.1126/science.1238477

[17] Taylor RA, White A, Sherratt JA 2013 How do variations in seasonality affect population cycles? Proc. R. Soc. B 280, 20122714 (doi:10.1098/rspb.2012.2714) 10.1098/rspb.2012.2714 PMC3574328 23325773

[18] McCann K, Hastings A, Huxel GR 1998 Weak trophic interactions and the balance of nature. Nature 395, 794–798. (doi:10.1038/27427)

[19] Mougi A, Nishimura K 2007 A resolution of the paradox of enrichment. J. Theor. Biol. 248, 194–201. (doi:10.1016/j.jtbi.2007.04.005) 17543997 10.1016/j.jtbi.2007.04.005

[20] Mougi A, Nishimura K 2008 Enrichment can damp population cycles: a balance of inflexible and flexible interactions. Oikos 117, 1732–1740. (doi:10.1111/j.1600-0706.2008.16688.x)

[21] Mougi A, Nishimura K 2008 The paradox of enrichment in an adaptive world. Proc. R. Soc. B 275, 2563–2568. (doi:10.1098/rspb.2008.0889) 10.1098/rspb.2008.0889 PMC2605811 18700201

[22] Mougi A, Nishimura K 2009 Imperfect optimal foraging and the paradox of enrichment. Theor. Ecol. 2, 33–39. (doi:10.1007/s12080-008-0026-0)

[23] Kingsland S. 1995 Modelling nature: episodes in the history of population ecology. Chicago, IL: University of Chicago Press.

[24] Huisman J, Weissing F 1999 Biodiversity of plankton by species oscillations and chaos. Nature 402, 407–410. (doi:10.1038/46540)

[25] Benincà E, Jöhnk KD, Heerkloss R, Huisman J 2009 Coupled predator–prey oscillations in a chaotic food web. Ecol. Lett. 12, 1367–1378. (doi:10.1111/j.1461-0248.2009.01391.x) 19845726 10.1111/j.1461-0248.2009.01391.x

[26] Mougi A, Kondoh M 2012 Diversity of interaction types and ecological community stability. Science 337, 349–351. (doi:10.1126/science.1220529) 22822151 10.1126/science.1220529

[27] Mougi A, Kondoh M 2014 Adaptation in a hybrid world with multiple interaction types: a new mechanism for species coexistence. Ecol. Res. 29, 113–119. (doi:10.1007/s11284-013-1111-4)

[28] Mougi A, Kondoh M 2014 Stability of competition-antagonism-mutualism hybrid community and the role of community network structure. J. Theor. Biol. 360, 54–58. (doi:10.1016/j.jtbi.2014.06.030) 25008419 10.1016/j.jtbi.2014.06.030

[29] Kondoh M, Mougi A 2015 Interaction-type diversity hypothesis and interaction strength: the condition for the positive complexity-stability effect to arise. Popul. Ecol. 57, 21–27. (doi:10.1007/s10144-014-0475-9)

[30] Mougi A. 2016 Stability of an adaptive hybrid community. Sci. Rep. 6, 28181 (doi:10.1038/srep28181) 27323666 10.1038/srep28181 PMC4914837

[31] Mougi A, Kondoh M 2014 Instability of a hybrid module of antagonistic and mutualistic interactions. Popul. Ecol. 56, 257–263. (doi:10.1007/s10144-014-0430-9)

[32] Toju H, Guimarães PR, Olesen JM, Thompson JN. 2014 Assembly of complex plant-fungus networks. Nat. Commun. 5, 5273 (doi:10.1038/ncomms6273) 25327887 10.1038/ncomms6273 PMC4218951

[33] Toju H, Guimarães PR, Olesen JM, Thompson JN. 2015 Below-ground plant–fungus network topology is not congruent with above-ground plant–animal network topology. Sci. Adv. 1, e1500291 (doi:10.1126/sciadv.1500291) 26601279 10.1126/sciadv.1500291 PMC4646793

[34] Karakashian SJ 1963 Growth of *Paramecium bursaria* as influenced by the presence of algal symbionts. Physiol. Zool. 36, 52–68. (doi:10.1086/physzool.36.1.30152738)

[35] Berger J 1980 Feeding behaviour of *Didinium nasutum* on *Paramecium bursaria* with normal or apochlorotic zoochlorellae. Microbiology 118, 397–404. (doi:10.1099/00221287-118-2-397)

[36] Vandermeer JH 1969 The competitive structure of communities: an experimental approach with Protozoa. Ecology 50, 362–371. (doi:10.2307/1933884)

[37] Ringel MS, Hu HH, Anderson G, Ringel MS 1996 The stability and persistence of mutualisms embedded in community interactions. Theor. Popul. Biol. 50, 281–297. (doi:10.1006/tpbi.1996.0032) 9000491 10.1006/tpbi.1996.0032

[38] Morris WF, Bronstein JL, Wilson WG 2003 Three-way coexistence in obligate mutualist-exploiter interactions: the potential role of competition. Am. Nat. 161, 860–875. (doi:10.1086/375175) 12858272 10.1086/375175

[39] Melián CJ, Bascompte J, Jordano P, Krivan V 2009 Diversity in a complex ecological network with two interaction types. Oikos 118, 122–130. (doi:10.1111/j.1600-0706.2008.16751.x)

[40] Fontaine C, Guimarães PR Jr, Kéfi S, Loeuille N, Memmott J, van der Putten WH, van Veen FJF, Thébault E 2011 The ecological and evolutionary implications of merging different types of networks. Ecol. Lett. 14, 1170–1181. (doi:10.1111/j.1461-0248.2011.01688.x) 21951949 10.1111/j.1461-0248.2011.01688.x

[41] Pocock MJO, Evans DM, Memmott J 2012 The robustness and restoration of a network of ecological networks. Science 335, 973–977. (doi:10.1126/science.1214915) 22363009 10.1126/science.1214915

[42] Sauve A, Thébault E, Pocock MJO, Fontaine C 2016 How plants connect pollination and herbivory networks and their contribution to community stability. Ecology 97, 908–917. (doi:10.1890/15-0132.1) 10.1890/15-0132.1 28792600

[43] Vance PR 1985 The stable coexistence of two competitors for one resource. Am. Nat. 126, 72–86. (doi:10.1086/284397)

[44] Bachelot B, Uriarte M, McGuire K 2015 Interactions among mutualism, competition, and predation foster species coexistence in diverse communities. Theor. Ecol. 8, 297–312. (doi:10.1007/s12080-015-0251-2)

[45] Barraquand F et al. 2017 Moving forward in circles: challenges and opportunities in modelling population cycles. Ecol. Lett. 20, 1074–1092. (doi:10.1111/ele.12789) 28633194 10.1111/ele.12789

