# Supplementary material for: Population cycles emerging through multiple interaction types
Source: R Soc Open Sci. 2017 Sep 27;4(9):170536. doi: 10.1098/rsos.170536 (PMC5627099; doi:10.1098/rsos.170536)
Supplement: Local stability analysis of the coexistence equilibrium [file rsos170536supp1.pdf]

Electronic supplementary material (S1)

Local stability analysis of the coexistence equilibrium

We examine the conditions for (1) the existence of nontrivial equilibrium with X^*, W^*, Y^*, $Z^* > 0$ and (2) local stability.

Existence of nontrivial equilibrium

By setting the right-hand sides of Eqs. 1a-d in the main text equal to zero, the nontrivial equilibrium is obtained as $X^* = d/ga$, $W^* = r_W - \alpha X^*$, $Y^* = (1/a)\{r_X - X^* - \beta W^* + uZ^*/(h_Z + Z^*)\}$ and $Z^* = r_Z + vX^*/(h_X + X^*)\}$.

The four-species equilibrium is feasible if and only if:

$$r_W - \alpha X^* > 0 \quad \text{[S1-1a]}$$

and

$$r_X - \beta r_W - (1 - \alpha\beta)X^* + uZ^*/(h_Z + Z^*) > 0 \quad \text{[S1-1b]}$$

Ineq. (S1-1) represents the condition for the competitor to exist at equilibrium. This requires weaker competitive ability of resource species and/or efficient exploitation of the resource species (large a; thus, small $X^* = d/ga$). It also suggests that even in stronger competitive ability of resource species, a sufficient strong exploitation of the resource species allows the competitor persist. Ineq. (S1-2) represents the condition for the exploiter to exist at equilibrium. This requires weaker competitive ability of competitor and/or efficient exploitation of the resource species. However, even when these conditions are not met, the exploiter can persist at equilibrium by a sufficient large

mutualistic effect (last term in l.h.s in (S1-2)). When the exploitation rate (a) is extremely small, the mutualistic effect converges to $u(r_Z + v)/(r_Z + h_Z + v)$, suggesting that the mutualism effect (large u and/or v) allows persistence of the exploiter, even if the exploitation rate is small.

Stability of the coexistence equilibrium

Even if the equilibrium is feasible (X^*, W^*, Y^*, $Z^* > 0$), the four species may not stably coexist because the equilibrium might not be stable. In this section, using a local stability analysis, we examine the stability of the coexistence equilibrium. The local stability of the system described by Eq. (1) in the main text is analyzed by linearizing the dynamics near the nontrivial equilibrium. We can judge the local stability by whether the characteristic equation of their Jacobian matrix satisfies the Routh-Hurwitz criteria. Under the equilibrium condition, we obtain the Jacobian matrix,

$$J = \begin{pmatrix} r_X - \alpha W^* - 2X^* - aY^* + \dfrac{uZ^*}{h_Z + Z^*} & -aX^* & \dfrac{h_Z u X^*}{\left(h_Z + Z^*\right)^2} & -\alpha X^* \\ agY^* & -d + agX^* & 0 & 0 \\ \dfrac{h_X v Z^*}{\left(h_X + X^*\right)^2} & 0 & r_Z + \dfrac{vX^*}{h_X + X^*} - 2Z^* & 0 \\ -\beta W^* & 0 & 0 & r_W - 2W^* - \beta X^* \end{pmatrix}$$

The characteristic equation for determining the eigenvalues is $\lambda^4 + \omega_1 \lambda^3 + \omega_2 \lambda^2 + \omega_3 \lambda + \omega_4 = 0$, where

$$\omega_1 = X^* + Z^* + W^*, \qquad \text{[S1-2a]}$$

$$\omega_2 = daY^* + W^*Z^* + X^*\left[W^*(1-\alpha\beta) + Z^*\left\{\frac{h_X h_Z uv}{(h_X + X^*)^2 (h_Z + Z^*)^2}\right\}\right], \qquad \text{[S1-2b]}$$

$$\omega_3 = daY^*(Z^* + W^*) + X^*Z^*W^*\left\{1 - \alpha\beta - \frac{h_X h_Z uv}{(h_X + X^*)^2 (h_Z + Z^*)^2}\right\}, \qquad \text{[S1-2c]}$$

$$\omega_4 = daY^*Z^*W^*, \qquad \text{[S1-2d]}$$

The equilibrium point is locally stable if ω_1, ω_3, $\omega_4 > 0$ and $\omega_1\omega_2\omega_3 > \omega_3^2 + \omega_1^2\omega_4$, according to the Routh-Hurwitz criteria. Because ω_1, $\omega_4 > 0$, the stability condition reduces to two conditions, $\omega_3 > 0$ and $\omega_1\omega_2\omega_3 > \omega_3^2 + \omega_1^2\omega_4$. Since the last condition is too complicated to understand, we can show the condition, $\omega_3 > 0$ and a sufficient condition for instability in the last condition ($\omega_2 < 0$) (see Ineqs.(4) in the main text). In an extreme case where *a* is very large, the system is always unstable because $\omega_1\omega_2\omega_3 = \omega_3^2 + \omega_1^2\omega_4$.

The stability condition of AC module can be also analyzed. The characteristic equation for determining the eigenvalues is $\lambda^3 + \omega_1\lambda^2 + \omega_2\lambda + \omega_3 = 0$, where

$$\omega_1 = X^* + W^*, \qquad \text{[S1-3a]}$$

$$\omega_2 = daY^* + W^*(1-\alpha\beta), \qquad \text{[S1-3b]}$$

$$\omega_3 = daY^*W^*, \qquad \text{[S1-3c]}$$

The equilibrium point is locally stable if ω_1, $\omega_3 > 0$ and $\omega_1\omega_2 > \omega_3$, according to the Routh-Hurwitz criteria. Because ω_1, $\omega_3 > 0$, the stability condition reduces to $\omega_1\omega_2 > \omega_3$. Then we obtain the condition (Ineqs.(3)) shown in the main text.
