# Supplementary material for: Population cycles emerging through multiple interaction types
Source: R Soc Open Sci. 2017 Sep 27;4(9):170536. doi: 10.1098/rsos.170536 (PMC5627099; doi:10.1098/rsos.170536)

Electronic supplementary material (S2)

Supplemental figure

Figure S2: Parameter regions indicating stability of the equilibrium. Coexistence is impossible in black region; the coexistence equilibrium is stable in the white region; and the coexistence equilibrium is unstable (a limit cycle occurs) in the grey region. The values of competition coefficients are shown at upper side of the panels. Parameter values are same as in Fig. 1.

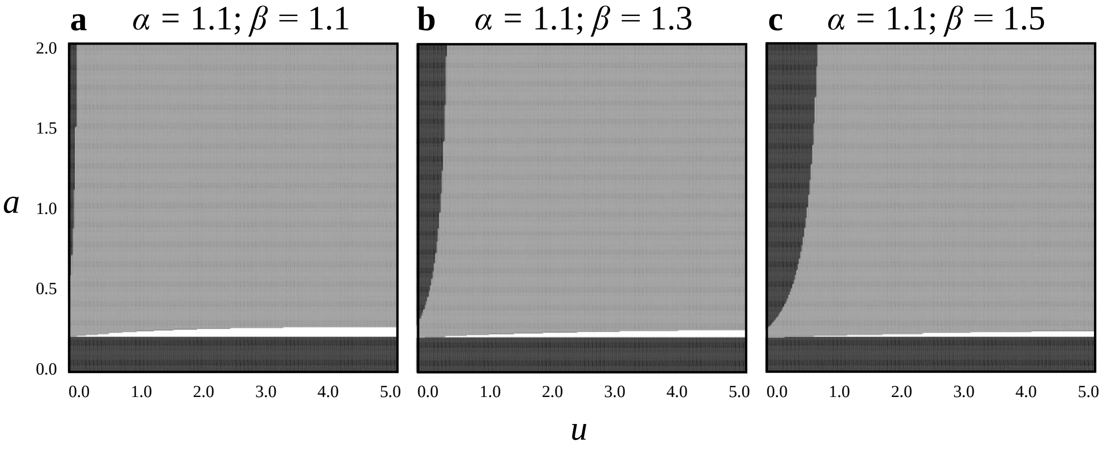

Supplement: Supplemental figure [file rsos170536supp2.pdf]
